# Thoracolumbar partial lateral corpectomy for the treatment of chronic intervertebral disc disease in 107 dogs

**DOI:** 10.1186/s13620-015-0056-z

**Published:** 2015-12-01

**Authors:** François-Xavier Ferrand, Pierre Moissonnier, Aurélie Filleur, Thibaut Cachon, Didier Fau, Eric Viguier, Claude Carozzo

**Affiliations:** Department of Small Animal Surgery, VetAgro Sup, Campus Vétérinaire de Lyon, Université de Lyon, Marcy L’Etoile, France; Department of Surgery, Veterinary Teaching Hospital, Veterinary School of Maisons Alfort, 94704 Maisons Alfort cedex, France; Department of Small Animal Surgery, VetAgro Sup, Campus Vétérinaire de Lyon, Université de Lyon, Marcy L’Etoile, France; Department of Small Animal Surgery, VetAgro Sup, Campus Vétérinaire de Lyon, Université de Lyon, Marcy L’Etoile, France; Unité ICE UPSP 2011-03-101, VetAgro Sup, Campus Vétérinaire de Lyon, Université de Lyon, Marcy L’Etoile, France

**Keywords:** Partial lateral corpectomy, Chronic ventral intervertebral disc disease, Dog

## Abstract

**Background:**

The purpose of this retrospective study was to assess short-and medium-term outcomes in dogs with chronic ventral thoracolumbar intervertebral disc disease (IVDD) treated by thoracolumbar partial lateral corpectomy (TLPLC).

Dogs surgically treated for chronic ventral IVDD by TLPLC were included. For each dog, neurological status evolution and complications were reported. Factors that could have influenced neurological recovery were statistically tested.

**Results:**

A total of 107 dogs were included in the study. Before surgery, 67.3 % of the dogs were able to walk, 24.3 % were grade 3, and 8.4 % were grade 4. The median hospitalization time was 3 days, and 82.2 % of the dogs were able to walk at discharge. The medium-term neurological grade was reached at a median time of 2 months. At the medium-term follow-up (median 12 months), 74.3 % of the dogs were neurologically improved, 22.9 % were stable, and 2.8 % were worsened. A total of 91.4 % of dogs were ambulatory, with 58.6 % of dogs having a normal gait. Preoperative neurological grade was significantly associated with the neurological status 24 h after the surgery and at discharge. Dogs with a higher preoperative neurological grade had a better chance of improving but lower odds of walking at 24 h after the surgery and at discharge compared with dogs with a lower preoperative grade. Spinal compression recurrence at the same surgical site was confirmed in 8 cases.

**Conclusion:**

Even if TLPLC leads to several intra and postoperative complications, this technique is a viable surgical option to treat chronic ventral IVDD.

**Electronic supplementary material:**

The online version of this article (doi:10.1186/s13620-015-0056-z) contains supplementary material, which is available to authorized users.

## Background

Thoracolumbar Partial Lateral Corpectomy (TLPLC) is one of the standard options considered in the treatment of chronic (protrusive or extrusive) thoracolumbar intervertebral disc disease (IVDD) [1, 2]. Initially described in 2004 in the dog [3], this technique, firstly defined “thoracolumbar lateral corpectomy”, involves the partial removal of thoracic or lumbar adjacent vertebral bodies that support the extruded/protruded disk material inside the vertebral canal. Flegel et al. reported that 90 % of patients demonstrated satisfactory spinal cord decompression after surgery and that the initial recommendations in the slot dimensions (25 % of body length, 50 % of body height, and 50–66 % of body width) do not lead to vertebral instability in clinical cases [4]. Biomechanical analysis showed a significant increase in range of motion during flexion/extension (32 %) and lateral bending (32 %) after TLPLC, but the authors concluded that stabilization might not be necessary [5]. In another study [6], the authors recommended combining mini-hemilaminectomy with TLPLC if necessary but to avoid hemilaminectomy without spinal stabilization. Successive lateral corpectomies can be performed in the same patient [4].

Only a few studies dealt with the treatments and outcomes of chronic IVDD in dogs [2, 3, 7–9]. Moreover, these studies included only a limited number of dogs.

Salger et al. [2] presented the neurological outcomes of 72 dogs treated by thoracolumbar TLPLC. Thirty-one of the 72 dogs demonstrated chronic IVDD in this study. The overall results did not differentiate between acute and chronic IVDD. Therefore, the aim of this bi-institutional study was to assess short-term and medium-term outcomes in 107 dogs presented with chronic IVDD and treated with TLPLC and to determine factors that could influence the outcome.

## Methods

Each patient’s owner was informed on the disease and the surgical technique used and accepted the risks of the anaesthesia and the surgical intervention. Pain was appropriately managed for each dog according to a pain score until the discharge.

### Case selection

Medical records (February 1996–March 2011) of dogs suffering from chronic IVDD and treated by TLPLC in 2 veterinary teaching hospitals were reviewed. Thoracolumbar IVDD was defined as chronic when dogs presented neurological signs, including pain, for 3 weeks or more. Moreover, IVDD was defined as chronic if disc protrusion was present and observed during the surgery or if the extrusive material showed adherences with the dura mater, the dorsal longitudinal ligament or the venous sinuses. Both criteria had to be fulfilled to define an IVDD as chronic.

Dogs were followed up at least for 6 months postoperatively for medium-term evaluation. Thoracolumbar partial lateral corpectomy was used to treat chronic ventral and ventro-lateral IVDD. The location of compression was assessed with myelography, CT myelography, or MRI. The data collected for each dog included the following: age, sex, breed, breed type (chondrodystrophic or non-chondrodystrophic), the duration of clinical signs before surgery, the assessment of preoperative neurological status, diagnostic imaging modalities used, affected intervertebral discs, postoperative assessments of the neurological status at 24 h after the surgery, at discharge, and at medium-term follow-up (at least 6 months postoperatively), postoperative complications, and the owners’ satisfaction. Dogs having several herniated discs with at least one ventral chronic compression were also included: in those dogs ventral compressions were treated by TLPLC and lateral compressions were treated by another technique like hemilaminectomy. Dogs were excluded from the study if there was a lack of follow-up data.

### Neurological assessment

The neurological status was classified from 0 to 5 [10] according to the clinical examination: grade 0 = normal; grade 1 = thoracolumbar pain without neurological deficit; grade 2 = rear limb ataxia, conscious proprioceptive deficit in rear limbs and ambulatory paraparesis; grade 3 = non-ambulatory paraparesis; grade 4 = paraplegia with or without bladder control, intact deep pain sensation; grade 5 = paraplegia, urine retention or overflow, and deep pain sensation loss.

The diagnosis and location identification of suspected lesions were achieved using clinical signs and myelography, CT scanning, or MRI.

### Surgical technique

Surgeries were performed by board-certified surgeons or by a resident under the direct supervision of one of the surgeons. Partial lateral corpectomy was performed as described by Moissonnier et al. [3] and was sometimes associated with other techniques performed at the same site: i) hemilaminectomy, ii) mini-hemilaminectomy or iii) foraminotomy. The lateral approach of the vertebrae was either a classic open approach [3] or an endoscope-assisted lateral corpectomy [11]. Any haemorrhage was controlled with pieces of muscle, bone wax or haemostatic sponges, depending on the origin of the bleeding (bone or venous sinus). Excision of the protruded disc or chronic extruded disc was considered complete when no further material could be collected from the site and when the spinal cord was returned to its normal position. Immediate post-operative pain was controlled by the administration of morphine and/or gabapentin. All the dogs received injections of morphine chlorhydrate either SC or IV at 0.1 to 0.3 mg/kg or at a constant rate infusion at 0.05 to 0.1 mg/kg/h according to the pain score and until the discharge. Gabapentin (10 to 40 mg/kg/d) was added according to the anaesthetist’s preference. Some dogs were administered anti-inflammatory drugs, either non-steroidal (meloxicam 0,1 mg/kg /d or carprofene 4 mg/kg/d) or steroidal (prednisolone, 0.1 mg/kg/d and then progressive dosage regression when used over 15 days) for a short period of time (5 to 15 days postoperatively). Neurological examinations were performed twice daily until discharge. Dogs were discharged if they did not demonstrate pain, if their neurological condition was not dramatically worsened, and if voluntary micturition control was present or manual urine expression from the bladder was easily achieved by palpation of the abdominal wall by the owners. Ambulation was not a condition for discharge.

### Follow-up

The neurological grade was evaluated 24 h after the surgery and at discharge. A medium-term evaluation (at least 6 months post surgery) was performed by a telephone interview of the owner or by mail. This evaluation was based on a questionnaire (see Additional file 1). The owners were asked about their level of satisfaction concerning the surgical intervention and the care provided to their animal. The results were reported as a number between 0 and 5; 0 indicated that they were not satisfied at all, and 5 indicated that they were completely satisfied.

For each clinical evaluation or using the owner questionnaire, the neurological status of each dog was classified as “improved” if it gained at least 1 neurological grade from the presurgical grade, “stable” if at the same neurological grade than before TLPLC, and “worsened” if they lost at least 1 neurological grade from the presurgical grade.

### Statistical analysis

The analyses were performed with R 3.1.1 (R Development Core Team 2014). The dependent variables evaluated were neurological progression (worsened, stable, improved) and the ability to walk (yes/no) at each follow-up time as well as the postoperative hospitalization length and the amount of time to reach the last grade evaluated. The independent variables tested for the association with dependent variables were as follows: the preoperative neurological grade, the duration of neurological dysfunction before the surgery, the number of chronic IVDD lesions treated by TLPLC for each dog during the same intervention, other IVDD lesions not treated by TLPLC due to a more lateral location of the disc hernia (yes or no), and the surgeon’s first-hand experience (Diplomate or resident). The independent variables were tested for correlations with Fisher’s exact test (two categorical variables), Spearman’s rank correlation (two numeric variables) or generalized linear regression (one numeric variable and one categorical variable). To evaluate the relationship between independent and dependent variables, either Fisher’s exact test, logistic regression or generalized linear regression was used. Independent variables were then tested in univariate analyses. Variables significantly associated with the dependent variables were then tested together in multivariate analyses. *P*-values <0.05 were considered significant.

## Results

### Signalment and duration of clinical signs before the surgery

**Fig. 1:**
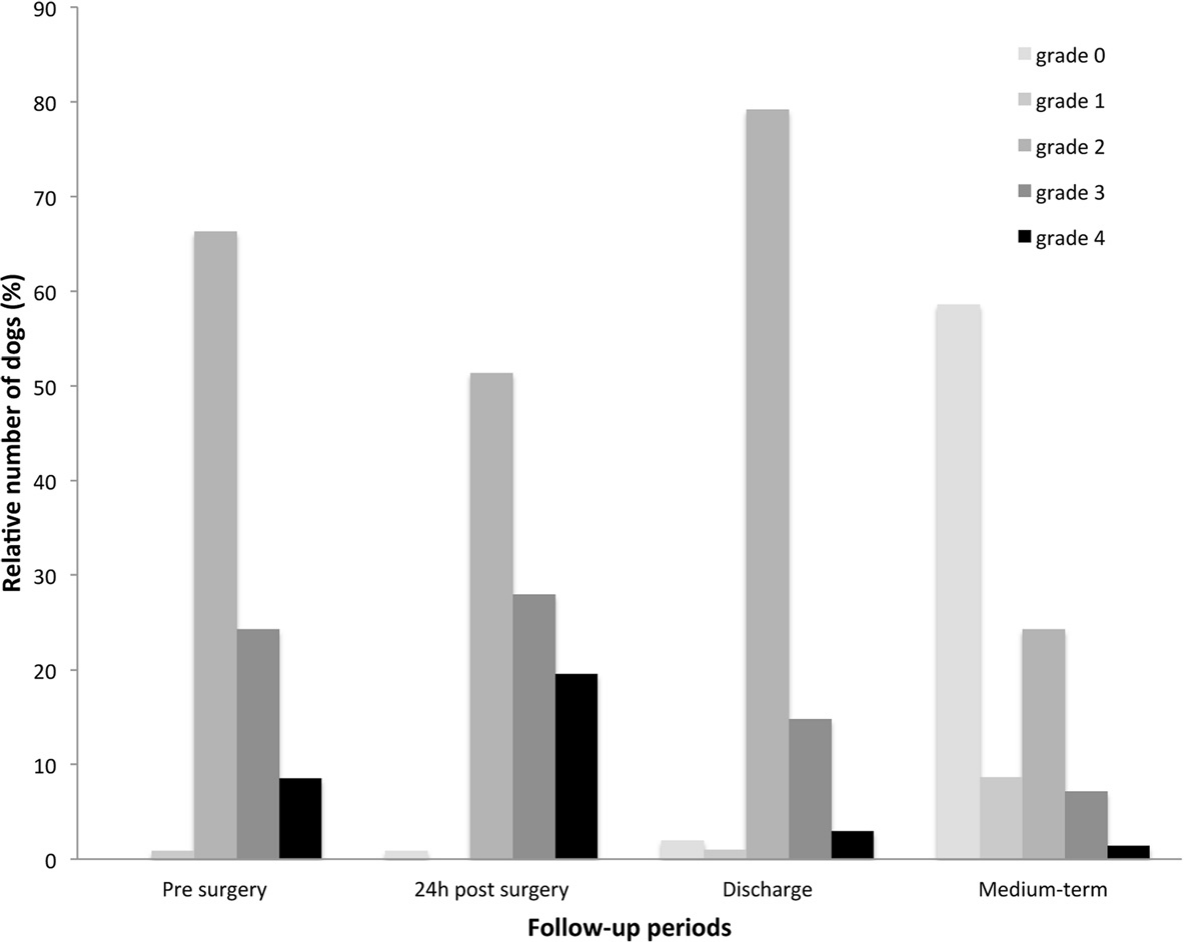
Evolution of neurological grades before and after treatment of IVDD by TLPLC in 107 dogs. Grade 0 = normal; grade 1 = thoracolumbar pain without neurological sign; grade 2 = ataxia, conscious proprioceptive deficit and ambulatory paraparesis; grade 3 = non-ambulatory paraparesis; grade 4 = paraplegia with or without bladder control, intact deep pain sensation; grade 5 = paraplegia, urine retention or overflow, and deep pain sensation loss

A total of 107 dogs were included in the study. The most frequently observed breed was German shepherd (23, 21.5 %). The mean age at the time of surgery was 8.4 ± 2.6 years. There were 28 females and 79 males. There were 41 chondrodystrophic dogs and 66 non-chondrodystrophic dogs. The median duration of clinical signs before the surgery was 3.5 months (range from 3 weeks to 78 months). Before surgery, 72 dogs (67.3 %) were ambulatory (1 dog grade 1, 71 dogs grade 2), whereas 35 dogs (32.7 %) were non-ambulatory (26 dogs were grade 3, 9 dogs were grade 4) (Table 1 and Fig. 1). There were no grade 5 dogs preoperatively.

**Table 1:** Neurological status of the dogs at the different follow-up periods after TLPLC for the treatment of chronic ventral IVDD

	24 h	Discharge (median 3 days)	Medium-term follow-up (at least 6 months postoperatively, median 12 months)
Improved	14/107 (*13.1* %)	24/101 (*23.8* %)	52/70 (*74.3* %)
Stable	60/107 (*56.1* %)	69/101 (*68.3* %)	16/70 (*22.9* %)
Worsened	33/107 (*30.8* %)	8/101 (*7.9* %)	2/70 (*2.8* %)
Ability to walk	56/107 (*52.3* %)	83/101 (*82.2* %)	64/70 (*91.4* %)

### Diagnostic imaging and surgery

Intervertebral disc herniation was diagnosed using standard radiographs and myelography procedures (67 dogs), MRI (22 dogs), CT myelography (16 dogs), or CT scanning and MRI (2 dogs). The most frequently affected intervertebral space by chronic ventral disc herniation was T13-L1 (n = 44; 27.5 %). A total of 160 IVDD lesions were treated with TLPLC, and 52 IVDD lesions in 52 dogs were treated with TLPLC combined with a hemilaminectomy (31 dogs), a mini-hemilaminectomy (15 dogs) or a foraminotomy (6 dogs) at the same site. Fourteen dogs had lateral spinal compressions on other sites and were treated with a technique other than TLPLC. Twenty-eight TLPLC procedures were endoscopically assisted. An experienced surgeon (Diplomate ECVS) was the first-hand surgeon in 58 % of cases, and a resident under direct supervision by an experienced surgeon was the first-hand surgeon in 13 % of cases. The experience level of the surgeon was not specified in 31 cases.

### Outcomes

Twenty-four hours after the surgery, 14 dogs were improved (13.1 %), 60 were stable (56.1 %), and 33 were worsened (30.8 %) (Table 1). Fifty-six dogs (52.3 %) were able to walk unassisted. Of the 35 dogs that were non-ambulatory before the surgery (grades 3 and 4), 10 dogs (28.5 %) regained the ability to walk 24 h after surgery. Of the 72 initially ambulatory dogs (grade 1 and 2), 46 dogs were still ambulatory 24 h after surgery (Fig. 1).

The median duration of hospitalization was 3 days (range 1 to 22 days). Six dogs died or were euthanized during hospitalization. Three were euthanized because of a worsening neurological status. The other three died for reasons unrelated to the neurological disease. All the dogs received morphine chlorhydrate after the surgery. Seven dogs received gabapentin, 77 dogs had a steroidal medication and 9 dogs had non-steroidal medication for 5 to 15 days postoperatively. At discharge, 24 dogs were improved (23.8 %), 69 dogs were stable (68.3 %), and 8 dogs were worsened (7.9 %) (Table 1). A total of 83 dogs (82.2 %) were ambulatory (grade 0 to 2) (Fig. 1).

Medium-term evaluation could be conducted for 70 dogs; 3 dogs died of unrelated reasons before 6 months post surgery, 5 dogs were euthanized because of the recurrence of neurological deficits before the 6-month follow-up, and 23 dogs were lost to follow-up. The median follow-up time was 12 months (range from 6 to 18 months), and the median time to reach the medium-term follow-up grade was 2 months (range from 1 day to 2 years). At the medium-term follow-up, 91.4 % of the dogs (64/70) were ambulatory, 52 dogs were neurologically improved (74.3 %), 16 were stable (22.9 %), and 2 were worsened (2.8 %) (Table 1, Fig. 1). There were no grade 5 dogs postoperatively.

The mean satisfaction level of the owners was 4.3/5 (median value of 5).

### Statistical analysis

Pairwise correlation tests between independent variables showed a significant relationship between the duration of clinical signs before the surgery and the number of chronic IVDD lesions treated by TLPLC (linear regression: *p* = 0.008), and a significant relationship between the preoperative grade and whether other IVDD lesions were treated by other methods: a higher preoperative grade was significantly associated with an increased chance that additional IVDD lesions were treated by other methods (Fisher’s exact test: *p* = 0.048). No other correlation was observed. For the purpose of statistical analysis, preoperative grades 1 and 2 (ambulatory dogs) were grouped at 24 h after surgery, at discharge and at medium-term follow-up due to the low number of dogs with grade 1, and preoperative grades 3 and 4 (non-ambulatory dogs) were grouped at discharge and at medium-term follow-up due to the low number of dogs with grade 4.

The preoperative grade had a significant effect on neurological evolution at 24 h (Fisher’s exact test: *p*-value < 0.001). A greater proportion of dogs with preoperative grades 3 and 4 were improved at 24 h compared with dogs with preoperative grades 1 and 2 (Table 2). Dogs with preoperative grades 3 and 4 had significantly lower odds of walking at 24 h than dogs with grades 1 and 2 (Table 3).

**Table 2:** Relative number of dogs distributed by preoperative grade and neurological evolution at 24 h and at discharge

		At 24 h postoperatively			At discharge (median 3 days)	
Preoperative grades		1&2	3	4	1&2	3&4
		(72 dogs)	(26 dogs)	(9 dogs)	(71 dogs)	(30 dogs)
Neurological evolution at 24 h post operatively (107 dogs)	Improved	2.8 %	34.6 %	33.3 %		
	Stable	59.7 %	42.3 %	66.6 %		
	Worsened	37.5 %	23.1 %	0 %		
Neurological evolution at discharge (101 dogs)	Improved				4.2 %	70 %
	Stable				84.5 %	30 %
	Worsened				11.3 %	0 %

**Table 3:** Relationship between the ability to walk at 24 h and preoperative grade (logistic regression)

		Odds ratio	Confidence interval		*P*-value
			2.5 %	97.5 %	
Preoperative grade (107 dogs)	Grade 1&2 (72 dogs)	-	-	-	-
	Grade 3 (26 dogs)	3.34	1.33	8.87	0.01
	Grade 4 (9 dogs)	14.15	2.41	270.16	0.01

The odds ratios are presented with their 95 % confidence interval and *p*-values obtained with a univariate logistic regression

There was no significant relationship between the hospitalization time and the variables tested (linear regression). The preoperative grade had a significant effect on neurological evolution at discharge (Fisher’s exact test: *p*-value < 0.001). A greater proportion of dogs with preoperative grades 3 and 4 became improved at discharge compared with dogs with preoperative grades 1 and 2 (Table 2). Univariate analyses of independent variables versus the ability to walk at discharge resulted in the selection of two variables: surgeon seniority and preoperative grade. By multivariate analysis, dogs treated by a senior surgeon had significantly higher odds of walking at discharge than those treated by a resident (Table 4). Dogs at grade 3 had significantly lower odds of walking at discharge than dogs at grades 1 and 2 (Table 4).

**Table 4:** Relationship between the ability to walk at discharge, the preoperative grade and the surgeon experience (logistic regression)

		Odds ratio	Confidence interval		*P*-value
			2.5 %	97.5 %	
Preoperative grade (101 dogs)	Grade 3 (23 dogs)	8.05	1.66	47.44	0.01
	Grade 4 (7 dogs)	4.56	0.16	74.05	0.29
Surgeon experience	Diplomate surgeon	0.14	0.02	0.69	0.02

The odds ratios are presented with their 95 % confidence interval and *p*-values obtained with a multivariate logistic regression

At medium-term follow-up, there was no significant relationship between neurological evolution, the ability to walk, or the amount of time required to reach the long-term grade and the variables tested (logistic and linear regression analyses).

### Complications

Twenty-five dogs (23.3 %) presented recurrent neurological deficits. Four of those dogs showed recurrence during the immediate postoperative period. The others dogs developed recurrences within a mean of 32 weeks (median 20 weeks, range 1.5–96 weeks). Eight dogs (7.4 %) were confirmed by either CT scan, MRI or ultrasound to have a compressive component at the same surgical site. A second surgery demonstrated that the compressive material was disc material in 7 dogs and a granuloma in one 1 dog. Four of these dogs also had other disc hernias on other sites. Two dogs were confirmed by MRI or CT scan to have disc hernias on other sites. Two dogs (1.8 %) developed vertebral instability after TLPLC (one with TLPLC alone and one with TLPLC and hemilaminectomy) that was demonstrated by a subluxation on standard x-rays or CT scan; one dog developed severe rear limb ataxia 15 days after the surgery and had surgical vertebral stabilization, and one dog showed rear limb ataxia 2 years after the surgery and was treated conservatively. Two dogs developed urinary and/or faecal incontinence during the immediate postoperative period that resolved in 5 and 6 months, respectively.

Minor complications included wound dehiscence (2 dogs), wound infection (3 dogs) and wound swelling with or without delayed healing (11 dogs). Vertebral ventral plexus haemorrhage was encountered in 26 (24.3 %) procedures. Six cases of nerve root damage (5.6 %) were documented, but none were associated with medium-term clinical signs. One dog presented a pneumothorax secondary to pleural puncture after TLPLC on T11-T12 intervertebral disc space.

## Discussion

We reported outcomes after TLPLC for the treatment of chronic ventral IVDD in 107 dogs. At the medium-term follow-up, 74 % of the dogs were improved, and 91 % were able to walk without assistance. We found neurological worsening in 30.8 % of our cases the day after TLLC. Neurological worsening has already been described after the treatment of chronic IVDD [8, 9]. In a study of Downes et al. [8], 17 dogs (60.7 %) showed a neurologic degradation 24 h after a hemilaminectomy and vertebral stabilisation for treatment of chronic disc protrusions, and 8 of the 17 dogs were ambulatory before the surgery.

We found that the preoperative grade had a significant effect on neurological evolution at 24 h after TLPLC and at discharge. This result must be cautiously interpreted because of the grading system used: the neurological improvement of dogs that were grade 2 could be underestimated because dogs that were classified as grade 2 could have shown inhomogenous neurological signs, ranging from mild conscious proprioceptive deficits to severe ambulatory parparesis. This is confirmed by the lack of significant association between the preoperative grade and long-term improvement.

Only a few studies have dealt with the treatment of chronic thoracolumbar IVDD in dogs [3, 7–9]. Although our results may be better than those already published [8, 9] comparison is difficult because of the low number of dogs included in these studies and variability of the procedures used to treat chronic IVDD.

In our study, 82.2 % of the dogs were able to walk at the end of the hospitalization, with a short mean hospitalization time of 3.6 days. Salger et al. [2] reported that 57.6 % of the dogs were able to walk unassisted at discharge (median time 4 days after surgery) after a partial lateral corpectomy. The difference between these studies can be explained by population differences: we included only chronic IVDD cases, with a high number of dogs with a low presurgical neurological grade, whereas in the study of Salger et al., the number of ambulatory dogs and the hospitalization time were calculated for all of the dogs (with both acute and chronic disc disease). Indeed, it is important to make the distinction between acute and chronic IVDD because the prognosis for recovery may be different with the same surgical technique. In chronic protrusive IVDD, the spinal cord is slowly compressed so that ischemia and degeneration of axons are expected. This physiopathology is different from the acute contusion encountered in acute extrusive IVDD [12].

Twenty-five dogs (23.3 %) presented with the recurrence of clinical signs after TLPLC. Only 8 (7.4 %) of them were confirmed with imaging diagnosis to have spinal cord compression at the same site. Salger et al. also reported that 18.3 % of the dogs had suspected or confirmed IVDD relapse, with one dog confirmed to have recurrence at the operated site [2]. Recurrence at the site of TLPLC is surprising because TLPLC theoretically allows for a more efficient power-assisted disk fenestration than manual disc fenestration, thus preventing the local recurrence of disc hernia [3, 13]. According to the surgical findings during the second surgery in the 8 dogs with local recurrence, residual compression was explained by inadequate removal of the IVD or formation of a compressive granuloma.

Overall 8 dogs (7.5 %) were euthanized between the surgery and the medium-term follow-up because of a undesired neurological outcome: 3 dogs for immediate postoperative worsening, and 5 dogs for recurrence of neurological signs. In these 5 dogs, the causative diagnosis had not been established.

Moissonnier et al. speculated that because of the anatomic structures in the region, other potential complications of the TLPLC included spinal nerve injury, vertebral sinus injury or pneumothorax risk in the thoracic region [3]. In our study, minor controllable haemorrhage of the vertebral sinus and nerve root damage occurred in 24.3 % and 5.6 % of the procedures, respectively. These results are comparable with those of the study by Salger et al. Haemorrhage of the vertebral sinus can be avoided if the drilling is stopped when the dorsal longitudinal ligament is reached, so that this ligament is situated between the instrument and the vertebral sinus, avoiding haemorrhage from the sinus and preventing iatrogenic injury to the spinal cord [3]. Thoracolumbar partial lateral corpectomy is known to induce some degree of vertebral instability [5, 6]. Two dogs in our study developed vertebral instability demonstrated by a vertebral subluxation on standard x-rays or CT scan. One dog had TLPLC alone and one had TLPLC combined with a mini-hemilaminectomy. Mini-hemilaminectomy can be combined with TLPLC to facilitate the removal of disc material and to provide a better visualization of the spinal cord without significantly destabilizing the vertebral column compared with TLPLC alone [6]. In contrast, the combination of TLPLC and hemilaminectomy is not recommended because this further destabilizes the spine and leads to permanent collapse of the intervertebral space [5, 6].

The major limitation of this study is its retrospective nature, similar to previous studies on chronic IVDD. Medium-term follow-up was based on either the owners’ perception or the referring veterinarian’s examination, which could lead to some degree of subjectivity or to a limitation in the reliability in the assessment of neurological function. The questionnaire sent to the owners could show several week points: some questions referred to a medical assessment like “walk unassisted” or “urinate normally” and it could be difficult for the owners to evaluate accurately those conditions. Moreover the questionnaire is a mixture of open and close questions so that it could be difficult for the owners to be constant and accurate in their answers. To palliate those week points, the questionnaire has been explained clearly to the owners by phone or during an intermediate consultation. Moreover some of the follow-ups were made by the referring veterinarian whose examination was more reliable.

Another limit was the definition of the 2nd grade in Scott’s classification [10]. This grade corresponds to pelvic limb paresis or ataxia in ambulatory dogs. This definition could overshadow the neurological improvement of a dog by calling it “stable”: for example, a preoperative grade 2 dog with postoperative significant improvement may still be classified in grade 2. That is a major limitation of most clinical studies assessing outcome after spinal surgery using a five point scale. Other grading systems should be used to overcome that obstacle in future prospective studies.

Finally, diagnostic imaging was lacking for the follow-up, especially in dogs that did not show a complete recovery. This could first have helped to determine if the lack of recovery was due to an incomplete decompression of the spinal cord, a technically induced injury, or a definitive lesion of the spinal cord secondary to chronic compression. Second, the quality of the decompression was not compared between the different surgeons; we found that dogs treated by a senior surgeon have significantly higher odds of walking at discharge than those treated by a resident, but we could not conclude whether the surgeon’s experience was important for the quality of the decompression. However, the experience level of the surgeon did not significantly influence the long-term outcome or the ability to walk. Visual assessment of the spinal cord and the vertebral plateau at the end of the surgery (the absence of a bulging plateau or disc and a straight spinal cord) are interesting criteria for spinal cord decompression but are certainly not as accurate as a post-operative CT scan or MRI.

## Conclusion

Ventral chronic IVDD treated with TLPLC resulted in 74 % of medium-term improvement with 91.4 % of the dogs able to walk unassisted. Recurrence of spinal compression at the same site was confirmed in 7.4 % of cases. Complications already described with TLPLC such as vertebral sinus haemorrhage and nerve root damage occurred in 24.3 % and 5.6 % of cases respectively, but without any clinical consequence. Despite those complications, TLPLC is a viable surgical option to treat chronic ventral IVDD.

## Additional file

Additional file 1:
**Questionnaire sent to owners to evaluate medium-term neurological status of the dogs after treating ventral chronic thoracolumbar IVDD by TLPLC, as well as the owner’s satisfaction.** (PDF 48 kb)
