# Supplementary material for: Thoracolumbar partial lateral corpectomy for the treatment of chronic intervertebral disc disease in 107 dogs
Source: Ir Vet J. 2015 Dec 1;68:27. doi: 10.1186/s13620-015-0056-z (PMC4666084; doi:10.1186/s13620-015-0056-z)
Supplement: Additional file 1: — Questionnaire sent to owners to evaluate medium-term neurological status of the dogs after treating ventral chronic thoracolumbar IVDD by TLPLC, as well as the owner’s satisfaction. (PDF 48 kb) [file 13620_2015_56_MOESM1_ESM.pdf]

Additional file 1: Questionnaire sent to owners to evaluate medium-term neurological status of the dogs after treating ventral chronic thoracolumbar IVDD by TLPLC, as well as the owner's satisfaction.

1. Neurological status

- Does your dog walk unassisted?
- If your dog did not walk at discharge from the hospital, when did it walk unassisted again?
- How would you qualify the gait of your dog? Normal, discrete stumbling and/or claws scraping on the floor, marked stumbling?
- If your dog cannot walk, does it have any movement of the hindlimbs?
- Does your dog urinate normally alone? Since when?
- Since when has your dog had its current neurological status?
- Do you think the neurological status of your dog has improved, worsened or remained stable since its discharge from the hospital?

2. Quality of life

- How would you quantity the quality of life of your dog?

Bad

1	2	3	4	5

Excellent

- Is your dog free of pain? Does it require pain medication?
- Does your dog require other medications?

3. Recurrence of clinical signs

- Has your dog presented recurrence of the same clinical signs as before the surgery? When?
- Has a diagnosis been made? How?
- What type of treatment has been realized? (surgical, medical)

4. Owners' satisfaction

- Are you satisfied with the medical care provided to your dog?

Not satisfied

0	1	2	3	4	5

Fully satisfied

- If you had a choice, would you make the same decision again?
